# The Impact of COVID-19 on Anxiety and Worries for Families of Individuals with Special Education Needs and Disabilities in the UK

**DOI:** 10.1007/s10803-021-05168-5

**Published:** 2021-07-01

**Authors:** V. Sideropoulos, D. Dukes, M. Hanley, O. Palikara, S. Rhodes, D. M. Riby, A. C. Samson, J. Van Herwegen

**Affiliations:** 1 Department of Psychology and Human Development, UCL, Institute of Education, University College London, London, UK; 2 Institute of Special Education, University of Fribourg, Fribourg, Switzerland; 3 Swiss Center for Affective Sciences, University of Geneva, Geneva, Switzerland; 4 Department of Psychology, Centre for Developmental Disorders, Durham University, Durham, UK; 5 Department for Education Studies, University of Warwick, Coventry, UK; 6 Centre for Clinical Brain Sciences, University of Edinburgh, Edinburgh, UK; 7 Faculty of Psychology, Unidistance Suisse, Brig, Switzerland

**Keywords:** COVID-19, Special Educational Needs and Disabilities (SEND), Anxiety, Worries, Wellbeing, Predictors

## Abstract

COVID-19 has affected people across the world. The current study examined anxiety and worries during the first UK national lockdown in March 2020. Parents (n = 402) reported on their own anxiety and worries as well as that of their son/daughter with Special Education Needs and Disabilities (SEND) and typically developing (TD) child (n = 186) at three time points. Although both groups showed increased anxiety across the three time points, levels of anxiety in the SEND group, but not the TD siblings, were predicted by awareness about COVID-19. In addition, worries differed between the groups showing that COVID-19 impacts the wellbeing of those with SEND differently to that of their TD siblings.

**Supplementary Information:**

The online version contains supplementary material available at 10.1007/s10803-021-05168-5.

The COVID-19 pandemic has had an overwhelming impact on the global population. Like other countries across the world, the UK has experienced a significant impact of COVID-19 on people’s lives. The first case was reported in the UK on January 31st, 2020 and, as of the 11th January 2021, nearly 12 months later, there have been 3,081,368 cases and 81,567 deaths in the UK alone (Johns Hopkins School of Medicine, 2021). On 23rd of March 2020 a national lockdown was announced in the UK which included Public health safety measures (PHSM) to reduce the spread of the virus (Cabinet Office, 2020). This lockdown was eased with a phased opening of schools on 5th of June 2020 and opening of shops on 15th of June (Institute for Government Analysis, 2021). During this time, schools closed for all but a few, including those with Special educational needs and disabilities (SEND), and people could only leave their premises for one hour on one occasion per day for activities such as physical exercise or shopping for essential goods, e.g., food, medicines. Different parts of the UK eased lockdown at different times, but generally Scotland, England, Wales and Northern Ireland have followed similar decision-making and time scales during the first strict lockdown. Since the end of the strict lockdown, a tiered system has been introduced with areas moving from a less restrictive way of living to again restrictive lockdowns at various times. This situation means that most children and young people’s lives as well as their family’s lives have been significantly disrupted by COVID-19.

Special educational needs and disabilities (SEND) is a broad term that refers to individuals that have difficulties and disabilities that can affect an individual's ability to learn and can arise from a wide range of factors. These can include having a developmental disorder diagnosis such as Autism spectrum disorder (ASD), Attention deficit hyperactivity disorder (ADHD), or Dyspraxia/Developmental coordination disorder (DCD) that impact learning or a more specific learning difficulty such as dyslexia, dysgraphia or dyscalculia. Individuals who are diagnosed with one of those conditions often show co-occurrences and at times most individuals with SEND meet criteria for more than one disorder. SEND may also arise from early adversity, prematurity or birth trauma. They may also arise from having a chromosomal disorder such as Down or Williams syndrome. In addition, there is a wide range of rare disorders with intellectual impairments for which the aetiology is currently unknown.

Reports have suggested that individuals with SEND are disproportionally impacted by COVID-19 (Rose et al., 2020; Van Herwegen et al., 2020a, 2020b). Individuals with SEND require additional support, not only for their learning (Iovannone et al., 2003; Rix et al., 2009) but often also to manage day-to-day tasks such as dressing, feeding and personal hygiene, as well as behaviours (Van Herwegen et al., 2018, 2019). This support is often provided by a range of professionals within the school and the community. During the lockdown from March-June 2020, a significant number of these services were restricted and disrupted (Jeste et al., 2020; Rose et al., 2020), even for those children with SEND that could attend school during the COVID-19 pandemic (Van Herwegen et al., 2020a, 2020b). In addition, individuals with SEND often have intellectual disabilities that may impact on their understanding of significant events, e.g., COVID-19 pandemic, as a whole (Aishworiya & Kang, 2020) or the new social norms and rules related to such events, e.g., social distancing (Mutluer et al., 2020). Furthermore, a number of individuals with SEND are at an increased risk of anxiety and mental wellbeing difficulties (Becker et al., 2015; Harrowell et al., 2017; Nelson & Harwood, 2011; Wigham et al., 2017). Seeing that many of those with SEND often prefer and benefit from a strict and consistent structure (Lindsay et al., 2014) and may not understand the sudden new social rules or reasons behind them (Courtenay & Perera, 2020), COVID-19 disruptions and new social rules may have provided an additional challenge for them. Finally, many groups of individuals with SEND have physical health problems, including congenital heart problems, hypothyroidism, increased risk for pneumonia (Alexander et al., 2020) and thus parents have been reluctant to send their children with SEND to school because of the increased risk of infection (Toseeb et al., 2020). In sum, there are several aspects related to COVID-19 that may have impacted on the wellbeing of an individual with SEND and their families.

Individuals with SEND do not only experience difficulties that impact themselves, but these can also cause added stress for their caregivers (Asbury et al., 2020; Ashworth et al., 2019). Various studies report on the fact that families of individuals with SEND feel additional pressures compared to families of children with only typically developing (TD) children (Chafouleas et al., 2020; Dhiman et al., 2020; Neece et al., 2020; O’Hagan & Kingdom, 2020). The majority of the parents reported struggling to provide the main care and educational support for the individual with SEND in combination with their work and other childcare commitments. In addition, families reported still needing to receive external support and thus, relied on TD siblings to provide additional care as well (O’Hagan & Kingdom, 2020). Evidence from two different online surveys for parents of children with SEND in the UK suggested that during lockdown the families would have benefited from, among other things, appropriate educational activities set by school, specialist professional advice for parents, regular structured activities outside home and materials or ideas for school learning (Asbury et al., 2020; Van Herwegen et al., 2020a, 2020b).

Seeing the difficulties that have been experienced by both individuals with SEND and their caregivers as a result of COVID-19, it can be assumed that their quality of life is impacted upon. The “Quality-of-life” model proposed by Schalock and Verdugo (2002), includes eight domains: Social Inclusion, Physical Well-Being, Interpersonal Relations, Material Well-Being, Emotional Well-Being, Self-Determination, Personal Development, and Rights (Schalock, 1996). Reduced quality of life (especially reduced physical wellbeing, social inclusion and reduced interpersonal relations because of COVID-19), are likely to impact on an individual’s wellbeing. Wellbeing can be defined in terms of anxiety as well as worrying about various health and family related matters.

Anxiety is a reaction to a potentially stressful or dangerous situation leading to a physiological response and cognitive processes helping us to deal with the difficult situation. However, high levels of anxiety can impact day to day functioning and ability to cope. High levels of anxiety affect between 7 and 15% of people in the typical population (Biedel & Turner, 2005). Yet, in children anxiety can vary between 2.6% and 41.2% in the general population (Cartwright-Hatton et al., 2006) averaging around 27% (Costello et al., 2005). There is evidence that individuals with SEND are at an increased risk of mental health difficulties (Becker et al., 2015; Harrowell et al., 2017; Nelson & Harwood, 2011; Wigham et al., 2017). This risk is likely to reflect a combination of social factors (Cooper et al., 2017) and differences in cognitive functions (Rhodes et al., 2012) that are associated with mental health risk, such as difficulties in executive functions (Matthews et al., 2008).

Emerging evidence suggests heightened levels of anxiety and depression among TD children during the COVID-19 pandemic (Duan et al., 2020; Jiao et al., 2020). Brooks et al. (2020) conducted a review of psychological effects of being isolated during quarantine in the general population and found increased confusion, anger, frustration, boredom, financial loss, and even post-traumatic stress disorder. Orgilés and colleagues (2020), reported that 85.7% of parents surveyed mentioned changes in their TD child’s emotional state, including difficulty concentrating, boredom, irritability, restlessness, and nervousness and that there was a relationship between parents’ anxiety and the child’s reported increase in emotional symptoms. Feelings of loss and worry as well as changes in their behaviour have also been reported by parents of children with SEND during the initial phases of lockdown (Asbury et al., 2020). In addition, carers of both children and adults with intellectual disability had significantly greater levels of a wish fulfilment coping style, feelings of defeat/entrapment, anxiety, and depression compared to carers of children without intellectual disability (Chafouleas et al., 2020; Dhiman et al., 2020; Willner et al., 2020).

Although some studies have examined parental anxiety, little is known about how the mental health of individuals with SEND has been affected during these unprecedented times. Combined events of routine changes, school and day activity centre closures, confinement, reduced access to external professionals and interventions are likely to increase the mental health vulnerability of these individuals and their families (Aishworiya & Kang, 2020). There have been reports of increased symptoms amongst individuals with Obsessive Compulsive Disorder (Banerjee, 2020), autistic individuals (Mutluer et al., 2020), ADHD (Zhang et al., 2020), and anxiety disorders more generally (Kwong et al., 2020) during the COVID-19 period. According to O’Hagan and Kingdom (2020), 43% of those surveyed reported that their child with SEND showed increased anxiety at the start of COVID-19 and that their child was struggling with significant mental health problems. However, it is not clear which children are affected the most. For example, 38% of families reported that their child likes to live in their own world and that their child’s anxiety had improved during lockdown. In addition, levels of anxiety may also be affected by several individual factors such as age of the individual, gender, their overall health condition which may make them more at risk for COVID-19, their awareness of COVID-19 and whether the child has been diagnosed with an anxiety disorder. For example, it has been suggested that general anxiety disorder increases with age for individuals with Williams syndrome (Dodd et al., 2009; Lefeyer et al., 2006). Also, in general females are more likely to experience higher levels of anxiety and are more likely to be diagnosed with an anxiety disorder (McLean et al., 2011).

The current study is the first to our knowledge to examine the anxiety and worries of the entire family of individuals with SEND, including carers, their children with SEND as well as TD siblings during March to June 2020 in the UK. This will allow deeper examination into how the pandemic has affected individuals with SEND differently to TD siblings matched for familial situations and stressors. In addition, the current study did not only gather data about anxiety and worries at the time the parents or caregivers were filling out the survey, but also before the pandemic as well as at the beginning of the pandemic in the UK. This allowed examination of how anxiety and worries evolved as a direct result of the pandemic specifically.

The purpose of this paper is to explore the following specific research questions related to anxiety during three key time points using a cross-sectional design; time-point 1 before COVID-19/before March 2020 (Before), time-point 2 when COVID-19 started in March 2020 (Start), and time-point 3 when the survey was completed (Now), which was between April and June 2020 during national lockdown:

1. Whether there is an effect of time (in the three time-points) as well as group (individual with SEND versus TD sibling) on reported anxiety.
2. Which factors predict anxiety when COVID-19 started for individuals with SEND and TD population at the start of the pandemic?
3. How do worries change over the three time-points and how do these changes compare between the groups (individual with SEND versus TD sibling)?

Based on the previous literature discussed above, it was predicted for Research Question 1 that there would be an effect of time and group on the reported anxiety, in that anxiety levels would increase in both groups across the different time points but that individuals with SEND would have higher anxiety at all-time points. For Research Question 2, it was hypothesized that predictors such as age, overall health, gender, COVID-19 awareness, parental anxiety and (previous) anxiety diagnosis would predict anxiety levels for individuals with SEND and their TD siblings during the time that COVID-19 started, with those who were older, female, with poorer health, higher parental anxiety and an existing anxiety disorder as well as awareness of COVID-19 having higher reported anxiety. Lastly, related to worries, we hypothesized that both groups experience increased worries concerning a range of health, social, school and familial factors across the three time points. However, it was predicted that individuals with SEND would worry more, especially with regards to worries that relate to “changes” such as in social approach and daily structure (e.g., closure of school).

Examining the differences in reported anxiety between individuals with SEND and TD siblings as well as what aspects of wellbeing the two groups worry about, allows greater insight into how the pandemic has disproportionally impacted individuals with SEND as well as which individuals with SEND were affected most. This knowledge will be informative to further tailor interventions and access to support services.

## Methods

### Participants

Caregivers (5.72% male) of 402 individuals (64.25% male) with SEND completed the survey. These respondents were aged 21 to 73 years old (M = 45.47, SD = 9.48) and 235 (58.45%) had a university degree.

The individuals with SEND had a formal diagnosis of a range of learning and developmental difficulties as can be seen in Table 1 and about half of them (n = 209, 52%) had an additional diagnosis. The individuals with SEND ranged in age from 1 to 45 years old (M = 13.04, SD = 7.84). Almost all of the individuals with SEND (n = 390, 97%) lived at home during the COVID-19 pandemic, with the remaining 3% living in supported accommodation. Most of these individuals with SEND remained at home as schools and work placements closed during the pandemic and thus, they had their daily routines disrupted (see Table 2).

**Table 1:** Overview of diagnosis of children with Special education needs and disabilities (SEND)

Type of diagnosis	Frequency	Percent	Valid percent
Autism	143	35.57	35.66
Down syndrome	103	25.62	25.69
Williams syndrome	43	10.70	10.72
Other genetic syndrome	40	9.95	9.98
Intellectual disabilities	20	4.98	4.99
Attention deficit hyperactivity disorder	18	4.48	4.49
Other	13	3.23	3.24
Learning difficulties (Dyslexia)	8	1.99	2.00
Anxiety/Mental health	8	1.99	2.00
Speech and language disorder	3	0.75	0.75
Developmental coordination disorder	2	0.50	0.50
Missing	1	0.25	
Total	402	100.00

**Table 2:** Overview of daily routines for individuals with Special educational needs and disabilities (SEND) and Typically developed (TD)

	Frequency	Percent	Valid percent
Send			
At home with family	325	80.85	81.45
Mainstream school	30	7.46	7.52
Special education school	20	4.98	5.01
Other	11	2.74	2.76
Pre-school	6	1.49	1.50
College	6	1.49	1.50
Work in community/Volunteer sector	1	0.25	0.25
Missing	3	0.75	
Total	402	100.00	
TD			
At home with family	105	26.12	26.12
Mainstream school	39	9.70	9.70
Paid full time/Part time work	21	5.22	5.22
Other	14	3.48	7.33
Work in community/Volunteer sector	3	0.75	0.75
Working in protected environment	2	0.50	0.50
Pre-school	1	0.25	0.25
Special school	1	0.25	0.25
Missing	216	53.73	53.73
Total	186	100.00	

Most individuals with SEND had mild to severe intellectual disabilities (n = 306, 76.12%) and thus, a proportion of individuals with SEND (n = 123, 30.60%) were reported to not be aware of COVID-19. Although these individuals may not have understood that any changes to routine might be caused by COVID-19, they may still have experienced higher anxiety as a result of the current pandemic and thus we included them in our analyses about anxiety (but not about worries). About a quarter of individuals with SEND (n = 133, 25.62%) had been reported to have an anxiety disorder. A minority of individuals with SEN (n = 31, 7.71%) were thought to have been affected by COVID-19 but only two were tested and needed hospital treatment.

A sub-sample of the caregivers (n = 186, 46%) also reported about a TD sibling (45% male). The TD siblings had a similar age range (1–47 years, *M* = 14.84, *SD* = 8.96) but were slightly older than the individuals with SEND; *t*(397) = 33.179, *p* < 0.001. Most of the TD siblings (n = 159, 85%) lived at home with their family and a large proportion remained at home due to COVID-19 closures of school and workplaces (see Table 2).

Most of the TD siblings were healthy with only 30 TD siblings (16%) reporting any medical issues of which only 6 had severe to serious medical issues (3%). For a minority (n = 28, 15.05%) caregivers reported an anxiety disorder. Again, a minority of TD siblings were thought to have been affected by COVID-19 (n = 11, 5.91%) but none had been tested or needed hospital treatment. Most of the TD siblings (n = 170, 91.40%) were aware of COVID-19.

In terms of caregivers’ anxiety, those who only had a SEND child to provide care for exhibited higher anxiety at the start of the pandemic (*n* = *399, M* = *3.10, SD* = *1.18*) compared to those who also had a TD child (n = 179, *M* = *2.32, SD* = *1.21).*

### Materials

An anonymous survey was distributed using Qualtrics, an online survey tool, which contained a range of open-ended and closed questions over four sections. This survey was used in the context of a larger international collaboration (www.specialneedscovid.org) with over 10,000 families of individuals with SEND across the globe. Three of these sections were relevant to the current study. The entire survey can be accessed freely on the OSF website (Van Herwegen et al., 2020): https://osf.io/5nkq9/.

Section A asked questions about the respondent and their SEND child’s background, including demographic information such as age, gender, medical background related to anxiety disorders, medication, general medical conditions and diagnosis of the child with SEND.

Section B included questions about the timing of the events related to COVID-19, including when the child’s school closed, or their daily routine changed because of lockdown.

Section C focused on the worries of the participating caregiver, the worries of their child with SEND and (if they also had a typically developing child) the worries and wellbeing of a TD child. For each question, the caregiver was asked to provide an answer related to three different time points: before the pandemic began (Before), when COVID-19 first affected them in March 2020 (Start), and at the time the participant completed the survey (Now), which was between April and June 2020. To measure anxiety, caregivers were asked to identify on a scale from 1 to 5 (with 1 not anxious at all to 5 being very anxious) their own anxiety, the level of anxiety that their child with SEND experienced and the anxiety of the TD child (if they had one).

The thirteen questions around worries were informed by the wellbeing categories as defined by Schalok (1996) and included worries related Social Inclusion (e.g., not being able to meet others), Physical Wellbeing (e.g., worries about catching COVID-19 and own health), Interpersonal Relations (e.g., worry about family conflict and others becoming ill), Material Wellbeing (e.g., financial worries), Emotional Wellbeing (e.g., worries about boredom), Self-Determination (e.g., loss of routine), and Personal Development (e.g., loss of institutional support). These were grouped into the following categories: Health Related Worries, Social Related Worries, School Closure Related Worries and Family Related Worries. Caregivers were asked to rate SEND’s and TD’s worries on a scale from 1 to 5 (with 1 not concerned at all to 5 being very concerned).

### Procedure

Respondents were recruited through social networks, social media and by emails addressed to special education institutions and support groups throughout the UK, including Williams Syndrome Foundation, Down Syndrome Association UK, ADHD Foundation. In addition, flyers and posters were also made available via Twitter and Facebook support groups.

Caregivers completed the survey between 8th of April 2020 and 27th of June 2020.

They were not reimbursed for their time and participation was entirely voluntarily as well as anonymous.

### Ethics

Ethical approval for the study was obtained from Ethics Commission of UniDistance, Switzerland before the start of the study. Respondents provided written consent to take part in the online study.

## Results

### Anxiety

#### Effect of Time on Anxiety for Children with SEND and TD Children

**Fig. 1:**
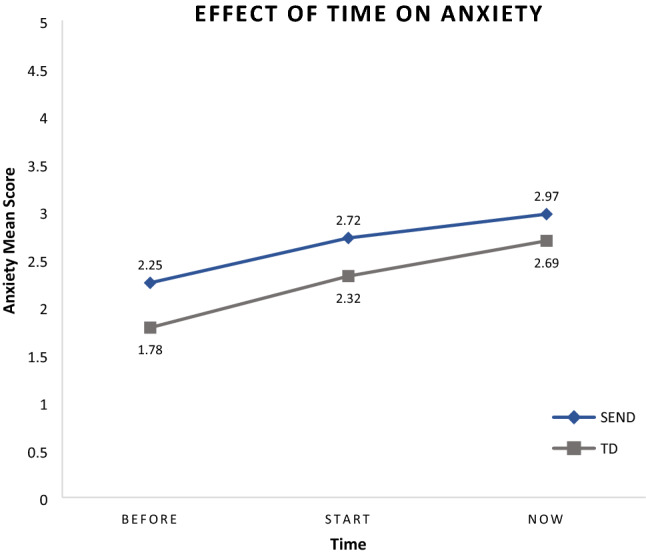
Overall Anxiety for SEND and TD groups at the three time points: Before (before March 2020), Start (from March 2020), Now (between April- June 2020)

To determine the effect of time on anxiety for children with SEND and TD children, a 3 (Time) × 2 (Group) mixed-model ANOVA was conducted. There was a significant main effect for Time. Mauchly’s Test of Sphericity indicated that the assumption of sphericity had been violated: χ^2^(2) = 73.61, *p* < 0.001. Therefore, the degrees of freedom had to be adjusted by using the Huynh–Feldt correction (ε = 0.89); *F*(1.79,1023.54) = 180.50, *p* < 0.001, η^2^_p_ = 0.24. This effect tells us that there was a difference in the reported anxiety for both groups over time. Post-hoc comparisons were computed and a difference between all time–points was evident (see Table S1 supplementary materials). As can be seen in Fig. 1, for both groups anxiety increased over time.

There was also a main effect for Group, *F*(1,572) = 13.12, *p* < 0.001, η^2^_p_ = 0.02, meaning there was a difference between the groups’ reported anxiety. Post-hoc analyses were conducted, individuals with SEND scored higher anxiety (M = 2.71) compared to the TD group (M = 2.33) (see Table S2 supplementary materials).

Our mixed model indicated that there was not a significant Time×Group interaction. Mauchly’s Test of Sphericity showed that the assumption of sphericity had been violated: χ^2^ (2) = 73.61, *p* < 0.001. The degrees of freedom had to be adjusted by using the Huynh–Feldt correction (ε = 0.89); *F*(1.79,1023.54) = 2.32, *p* = 0.10, η^2^_p_ = 4.04e-3.

### Predictors of Anxiety

#### Linear Regression to Predict Anxiety for SEND group

A multiple linear regression was run to predict anxiety at the start of the pandemic (time-point 2) from age, gender, health, whether they were aware of COVID-19 and whether they had a diagnosis of anxiety prior to COVID-19 as well as caregiver anxiety for the same time-point for the SEND group. Seeing that the aim of this analysis was to examine predictors of anxiety, we selected time-point 2 instead of time-point 3 as time-point 2 was the beginning of the pandemic and we use it as an uncertain stressful event that was more comparable between respondents compared to the time-point 3 when caregivers completed the survey.

The data was screened for assumptions and outliers and all assumptions of linearity, normality, homoscedasticity, and multicollinearity were found to be met. The multiple regression model statistically significantly predicted Anxiety levels at the beginning of the pandemic, *F(*6,388) = 49.55, *p* < 0.001, R2 = 0.41 and R2 adjusted = 0.40. Not all six variables added statistically significantly to the prediction. Regression coefficients (b and β) and standard errors along with 95% confidence intervals can be found in Table 3. There was no association between age (β = 0.06, *p* = 0.13), gender (β = 0.06, *p* = 0.11), health status (β = 0.04, *p* = 0.38) and the child with SEND’s anxiety at the start of the pandemic at time-point 2. However, caregiver anxiety (β = 0.18, *p* < 0.001), the child with SEND’s anxiety disorder (β = 0.46, *p* < 0.001) and the child with SEND’s COVID-19 awareness (β = 0.27, *p* < 0.001) significantly predicted Anxiety at time-point 2. SEND children of anxious caregivers were more likely to show higher anxiety as well as those who were diagnosed with an anxiety disorder and were aware of COVID-19 in the beginning of the COVID-19 pandemic.

**Table 3:** Coefficients for linear regression for SEND

Model		Unstandardized B	Standard error	Standardized β	t	*p*	95% CI		Collinearity statistics	
							Lower	Upper	Tolerance	VIF
1	(Constant)	− 0.71	0.40		− 1.79	0.08	− 1.49	0.07		
	Age	0.01	0.01	0.06	1.51	0.13	0.00	0.03	0.90	1.11
	Health status	− 0.05	0.06	− 0.04	− 0.89	0.38	− 0.18	0.07	0.94	1.07
	Caregiver reported anxiety (Time- point 2)	0.20	0.05	0.18	4.46	0.00	0.11	0.29	0.98	1.02
	Gender	− 0.18	0.11	− 0.06	− 1.58	0.11	− 0.39	0.04	0.99	1.01
	Anxiety disorder	1.32	0.12	0.46	10.87	0.00	1.09	1.56	0.85	1.17
	COVID- 19 awareness	0.80	0.13	0.27	6.30	0.00	0.55	1.05	0.83	1.21

#### Linear Regression to Predict Anxiety for TD group

A multiple linear regression with the same predictors used for the SEND model was computed but this time for the TD group’s anxiety during time-point 2 was analysed. All assumptions were met, and no outliers were detected. The regression model statistically significantly predicted Anxiety levels when the pandemic started, *F*(6,56) = 4.43, *p* < 0.001, R2 = 0.32 and R2 adjusted = 0.25. Associations between caregiver’s reported anxiety for time point 2 (β = 0.27, *p* < 0.02) and anxiety disorder (β = 0.35, *p* < 0.01) with Anxiety of TD individuals for time-point 2 were detected. But there was no association between age (β = 0.19, *p* = 0.10), gender (β = 0.03, *p* = 0.76), healthy status (β = − 0.15, *p* = 0.20), COVID-19 awareness (β = − 0.12, *p* = 0.31) with TD individuals’ Anxiety. Table 4 provides the regression coefficients (both beta and β) along with standard errors and the 95% confidence intervals of our model. TD children with anxious caregivers as well as TD children who were diagnosed with an anxiety disorder were more likely to show higher anxiety.

**Table 4:** Coefficients for linear regression for TD

Model		Unstandardized B	Standard error	Standardized β	t	*p*	95% CI		Collinearity statistics	
							Lower	Upper	Tolerance	VIF
1	(Constant)	1.87	1.69		1.11	.27	− 1.51	5.24		
	Age	0.03	0.02	0.19	1.66	.10	− 0.01	0.06	0.89	1.12
	Health status	− 0.25	0.19	− 0.15	− 1.30	.20	− 0.63	0.13	0.98	1.02
	Caregiver reported anxiety (Time- point 2)	0.27	0.11	0.27	2.36	.02	0.04	0.50	0.96	1.04
	Gender	0.08	0.27	0.03	0.31	.76	− 0.46	0.63	0.97	1.03
	Anxiety disorder	1.16	0.40	0.35	2.92	.01	0.36	1.96	0.86	1.16
	COVID- 19 awareness	− 0.56	0.55	− 0.12	− 1.02	.31	− 1.67	0.54	0.89	1.12

### Reported Worries

**Fig. 2:**
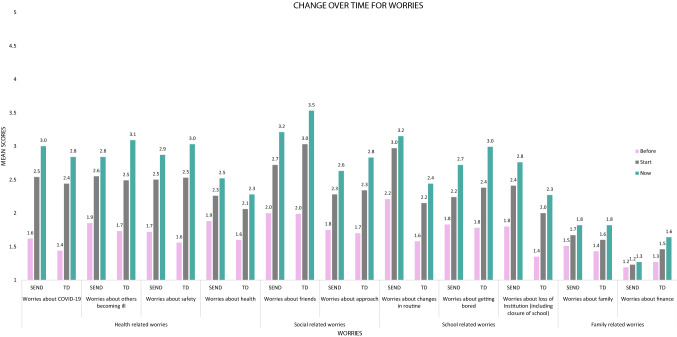
Change over time for worries

For the analyses related to worries, only those individuals with SEND (n = 279) and the TD siblings who were aware of COVID-19 (n = 173) were included in the repeated measures 3(Time)×2(Group) analyses, given that awareness of COVID-19 was an important predictor for anxiety in the SEND group. Sphericity violations for each model are presented in Table 5. The change over time for both SEND and TD for each type of worries is presented in Fig. 2. For a detailed overview of the mean scores for each category in the worries, see Table 6.

**Table 5:** Sphericity violations and ANOVA output for worries and concerns related to Wellbeing

Type of concern	Test of sphericity checks	Adjustment method and ε level	Source	d1	d2	F	p	η^2^_p_
Health-related worries								
Concerns about COVID-19	χ^2^ (2) = 27.37 *p* < .001	Huynh–Feldt correction (ε = .94)	Group	1	565	4.71	.03*	8.27e-3
			Time	1.87	676.11	174.24	< .001***	.33
			Group x Time	1.87,	676.11	0.14	.85	4.01e-4
Concerns about others becoming ill	χ^2^ (2) = 35.37, *p* < .001	Huynh–Feldt correction (ε = .92)	Group	1	360	0.03	.86	9.16e-5
			Time	1.84	661.43	153.08	< .001***	.30
			Group x Time	1.84	661.43	4.09	.02*	.01
Concerns about safety	χ^2^ (2) = 27.36, p < .001	Huynh–Feldt correction (ε = .94)	Group	1	362	6.37e-3	.94	1.76e-5
			Time	1.87	678.10	156.56,	< .001***	.30
			Group x Time	1.87	678.10	2.37	.10	6.52e-3
Concerns about health	χ^2^ (2) = 32.66, *p* < .001	Huynh–Feldt correction (ε = .92)	Group	1	361	2.97	.09	8.15e-3
			Time	1.85	667.63	74.49	< .001***	.17
			Group x Time	1.85	667.63	0.23	.78	6.27e-4
Social-related worries								
Concerns about friends	χ^2^ (2) = 55.44, *p* < .001	Huynh–Feldt correction (ε = .88)	Group	1	361	2.17	.14	5.97e-3
			Time	1.76	634.64	176.20	< .001***	.33
			Group x Time	1.76	634.64	3.32	< .004**	9.11e-3
Concerns about approach	χ^2^ (2) = 93.78, *p* < .001	Huynh–Feldt correction (ε = .82)	Group	1	362	0.25	.61	7.04e-4
			Time	1.63	591.49	102.57	< .001***	.22
			Group x Time	1.63	591.49	1.58	.21	4.36e-3
School-related worries								
Concerns about changes in routine	χ^2^ (2) = 37.94, *p* < .001	Huynh–Feldt correction (ε = .91)	Group	1	362	25.03	< .001***	.06
			Time	1.83	661.47	79.11	< .001	.18
			Group x Time	1.83	661.47	0.87	.41	2.41e-3
Concerns about getting bored	χ^2^ (2) = 85.18, *p* < .001	Huynh–Feldt correction (ε = .83)	Group	1	361	0.67	.41	1.86e-3
			Time	1.66	598.74	134.18	< .001***	.27
			Group x Time	1.66	598.74	3.03	.06	8.33e-3
Concerns about loss of institution (school closure)	χ^2^ (2) = 70.85, *p* < .001	Huynh–Feldt correction (ε = .85)	Group	1	360	9.15	< 2.66e-3***	.02
			Time	1.70	631.22	101.21	< .001***	.22
			Group x Time	1.70	613.22	0.17	.81	4.68e-4
Family-related worries								
Concerns about family	χ^2^ (2) = 121.75, *p* < .001	Huynh–Feldt correction (ε = .78)	Group	1	361	0.18	.67	4.99e-4
			Time	1.56	563.00	35.16	< .001***	.08
			Group x Time	1.56	563	0.52	.55	η^2^_p_ = 1.44e-3
Concerns about finance	χ^2^ (2) = 71.98, *p* < .001	Huynh–Feldt correction (ε = .85)	Group	1	359	7.96	< 5.05e-3***	.02
			Time	1.70	609.94	32.75	< .001	.08
			Group x Time	1	359	7.96	< 5.05e-3***	.02

**Table 6:** Sphericity violations and ANOVA output for worries related to Wellbeing

Type of concern	Test of sphericity checks	Adjustment method and ε level	Source	d1	d2	F	p	η^2^_p_
Health-related worries								
Worries about COVID-19	χ^2^ (2) = 27.37 *p* < .001	Huynh–Feldt correction (ε = .94)	Group	1	565	4.71	.03*	8.27e-3
			Time	1.87	676.11	174.24	< .001***	.33
			Group x Time	1.87,	676.11	0.14	.85	4.01e-4
Worries about others becoming ill	χ^2^ (2) = 35.37, *p* < .001	Huynh–Feldt correction (ε = .92)	Group	1	360	0.03	.86	9.16e-5
			Time	1.84	661.43	153.08	< .001***	.30
			Group x Time	1.84	661.43	4.09	.02*	.01
Worries about safety	χ^2^ (2) = 27.36, p < .001	Huynh–Feldt correction (ε = .94)	Group	1	362	6.37e-3	.94	1.76e-5
			Time	1.87	678.10	156.56,	< .001***	.30
			Group x Time	1.87	678.10	2.37	.10	6.52e-3
Worries about health	χ^2^ (2) = 32.66, *p* < .001	Huynh–Feldt correction (ε = .92)	Group	1	361	2.97	.09	8.15e-3
			Time	1.85	667.63	74.49	< .001***	.17
			Group x Time	1.85	667.63	0.23	.78	6.27e-4
Social-related worries								
Worries about friends	χ^2^ (2) = 55.44, *p* < .001	Huynh–Feldt correction (ε = .88)	Group	1	361	2.17	.14	5.97e-3
			Time	1.76	634.64	176.20	< .001***	.33
			Group x Time	1.76	634.64	3.32	< .004**	9.11e-3
Worries about approach	χ^2^ (2) = 93.78, *p* < .001	Huynh–Feldt correction (ε = .82)	Group	1	362	0.25	.61	7.04e-4
			Time	1.63	591.49	102.57	< .001***	.22
			Group x Time	1.63	591.49	1.58	.21	4.36e-3
Worries related to school closures								
Worries about changes in routine	χ^2^ (2) = 37.94, *p* < .001	Huynh–Feldt correction (ε = .91)	Group	1	362	25.03	< .001***	.06
			Time	1.83	661.47	79.11	< .001	.18
			Group x Time	1.83	661.47	0.87	.41	2.41e-3
Worries about getting bored	χ^2^ (2) = 85.18, *p* < .001	Huynh–Feldt correction (ε = .83)	Group	1	361	0.67	.41	1.86e-3
			Time	1.66	598.74	134.18	< .001***	.27
			Group x Time	1.66	598.74	3.03	.06	8.33e-3
Worries about the school or loss of institution	χ^2^ (2) = 70.85, *p* < .001	Huynh–Feldt correction (ε = .85)	Group	1	360	9.15	< 2.66e-3***	.02
			Time	1.70	631.22	101.21	< .001***	.22
			Group x Time	1.70	613.22	0.17	.81	4.68e-4
Family related worries								
Worries about family	χ^2^ (2) = 121.75, *p* < .001	Huynh–Feldt correction (ε = .78)	Group	1	361	0.18	.67	4.99e-4
			Time	1.56	563.00	35.16	< .001***	.08
			Group x Time	1.56	563	0.52	.55	η^2^_p_ = 1.44e-3
Worries about finance	χ^2^ (2) = 71.98, *p* < .001	Huynh–Feldt correction (ε = .85)	Group	1	359	7.96	< 5.05e-3***	.02
			Time	1.70	609.94	32.75	< .001	.08
			Group x Time	1	359	7.96	< 5.05e-3***	.02

#### Health-Related Worries

Worries about COVID-19 increased over time for both groups, but those with SEND were more concerned across all time points.

As can be seen in Fig. 2, the TD group scored higher worries about others becoming ill when the caregivers completed the survey (time point 3), while the SEND group showed higher worries when the COVID-19 pandemic started (time point 2) and their worries decreased afterwards.

While there was a difference in worries about Safety between time-points, this difference was not significant between the Groups. Similarly to other health worries, worries about Health increased with time, but there was no difference between the TD and SEND groups. These findings show that, although worries increased with time, the only group difference for health-related worries related to COVID-19 in general.

#### Social-Related Worries

As can be seen in Fig. 2, in both groups worries about friends increased over time but worries increased more in the TD group when the pandemic started and continued to increase over time. In contrast, worries related to approaching others (worries about Approach) increased with time in both groups, but there was not a difference between the TD and SEND groups.

#### Worries Related to School Closures

There were a number of group differences related to the school closures: There was an increase for worries about Change in Routine over time in both groups but those with the SEND reported higher worries at each of the time points. Also, in terms of “worries about the school closure or loss of institutional support”, both groups’ worries about school closure increased over time, but more so for those in the SEND group. Yet, there was no group difference related to worries about ‘getting bored’. As can be seen in Fig. 2, there is an increased worry about getting bored over time for both groups.

#### Family Related Worries

While there was an increase of worries about family over time (worries about family), both groups performed similarly. Yet, the TD group worried more about finances and their worries increased more over time compared to the SEND group.

## Discussion

The current study examined the impact of the early stages of COVID-19 in the UK on anxiety and worries of individuals with a wide range of SEND in relation to caregiver anxiety and compared to that of a group of TD siblings. In addition, predictors of anxiety were also examined for both the SEND and TD groups as well as what kind of aspects related to COVID-19 both groups worried about. Together these analyses allowed for a better understanding of the specific impact of COVID-19 on the wellbeing of individuals with SEND compared to TD populations and which individuals with SEND are most affected.

### Impact of COVID19 on Anxiety in SEND and TD groups

In terms of the first hypothesis and the overall reported anxiety and the effect of time, both groups increased over time but those with SEND scored higher on anxiety at all three time points, compared to the TD group. These findings are in line with previous studies that have reported high anxiety in those with special needs during the current pandemic (O’Hagan & Kingdom, 2020). However, the current study adds to previous studies, in that direct comparisons to siblings showed that anxiety tended to be higher across all time points for those with SEND. Finally, the current study shows that during stressful life events such as the current pandemic anxiety increases sharply for TD siblings as well.

### Anxiety Predictors for SEND and TD

For hypothesis 2, multiple regression analyses were run to explore what factors predicted anxiety at the start of the pandemic in March 2020. For the SEND group, higher levels of caregiver anxiety, a diagnosis of an anxiety disorder, and awareness of COVID-19 seemed to be strong predictors of anxiety during that time-point. Similar results were found for the TD group, with exception that awareness of COVID-19 was not a leading predictor of anxiety this time. Factors such as age, gender and health status did not seem to drive anxiety at all in contrast to our predictions. We discuss some of the limitations of the study below, but these findings suggest that predictors of anxiety during a pandemic might be different from general stressful events and thus replication and further investigation of what predicts anxiety during a pandemic, including factors not yet explored (e.g., IQ and social support), is required.

The present findings also suggest that anxiety increases in both groups and that there are few differences within the SEND and TD groups concerning which individuals are at greater risk or have heightened anxiety as a result of COVID-19. Ours is the first study to provide evidence that COVID-19 awareness can predict anxiety in individuals with SEND. Similarly, our study is in line with both Platt et al. (2016) and the recent study by Russell et al. (2020) which show that increased caregiver anxiety is linked with the severity of anxiety symptoms in their child, but also with the perceptions of children’s stress.

### Worries about the Impact of COVID-19 in SEND and TD Groups

In terms of hypothesis 3, both groups showed increased worries across the three time points for all types of worries. However, there were some interesting group differences and similarities. In line with the predictions, individuals with SEND worried more about COVID-19, whilst the TD group had a larger increase related to becoming ill more specifically. These differences could be explained by the fact that many individuals with SEND shielded^1^ (Van Herwegen et al., 2020a, 2020b) and those caregivers have been reluctant to send their children with SEND to school because of the increased risk of infection (Toseeb et al., 2020). As such, those with SEND might have been less worried about becoming ill but rather worried about COVID-19 more generally. Both groups showed increased worries related to general health but worries around safety decreased. The latter could be explained by the fact that most individuals in both groups remained at home during the lockdown.

Social related worries also increased in both groups, but the TD group showed a greater increase related to not being able to see friends. However, worries around social approach did not differ between the two groups and both groups showed increased worries over time. Finally, the TD group showed greater worries related to family related worries, especially finances.

In relation to school closures and closures of day or activity centres, both groups’ worries increased over the three time points. There were no differences between the two groups with regards to boredom. This finding is similar to studies in the USA related to TD individuals that reported that schools provide important structured activities that occupy TD individuals from becoming bored (Jeste et al., 2020; Orgilés et al., 2020). The current study showed that the same is true for those with SEND. However, the SEND group had higher levels of reported worries related to a lack of structure across the three time points and showed a greater increase of concern over the time points related to loss of support because of school and activity centres’ closures. This replicates the findings of O’Hagan and Kingdom (2020) but contrasting the worries of those with SEND to TD siblings, allowed further understanding of the unique worries of those with SEND.

### Limitations and Future Studies

The current study examined the worries and anxiety levels of individuals with SEND and TD siblings through caregiver report rather than through the voice of the individual with SEND or TD sibling themselves. Research has shown significant links between caregivers’ mental health and the way they perceive their children’s wellbeing as well as how caregivers’ mental health of disastrous events, e.g., COVID-19, can affect children’s understanding of such situations (Neece et al., 2012; Orgiles et al., 2020). Also, the current caregivers completed the survey during an ongoing pandemic and thus their recall of anxiety levels before the pandemic and at the start might have been affected. Therefore, there is some bias to be expected on the reported data of anxiety of the children.

In addition, although the current study examined for the first time several factors that may explain individual differences in each group related to the impact of COVID-19 on anxiety and worries, it was not possible to examine differences related to the primary diagnosis of the individuals with SEND because uneven groups were recruited and 70% of the individuals with SEND had one of the following developmental disorders: autism, Down syndrome or Williams syndrome, and some conditions were under-represented (e.g., socio-emotional and behavioral difficulties). However, previous studies that have examined outcomes in those with SEND have shown that categorical labels may not be informative with regards to the needs of the individual with SEND (Dockrell et al., 2019). Instead, examination of individual differences such as pre-existing health and anxiety diagnoses might be a more useful approach to examine those most affected or in need. Yet, our worldwide data of more than 10 000 families that completed the survey will allow further examination of individual differences related to health and physical needs as well as country specific variables (e.g., types of measures to deal with the pandemic, timing of restrictions, support available).

Finally, the current study used a cross-sectional design to address changes in anxiety for those with SEND and their TD siblings. However, these findings need to be replicated using longitudinal designs.

## Impact and Conclusion

Together, these findings show that COVID-19 impacts the worries of those with SEND differently to that of their TD siblings and that school closures have a particular effect on those with SEND. For individuals with SEND, schools and activity centers provide an important routine and structure that helps to reduce anxiety (Van Herwegen et al., 2020a, 2020b). In addition, schools and activity centers provide caregivers with access to specialist advice that would not only benefit individuals with SEND but also their caregivers’ anxiety (Asbury et al., 2020; Ashworth et al., 2019). Similarly, for the TD group, education provides a great opportunity for socialization with their peers, which consecutively could lead to reduced anxiety levels. The current study also showed that individuals with SEND who have a pre-existing diagnosis of an anxiety disorder and who are aware about COVID-19 are in greater need of interventions related to anxiety.

Awareness of COVID-19 is also a key predictor for SEND when looking at anxiety levels. Such evidence might be explained by the complexity of the current events and that the associated rules of these events might be more difficult to be comprehended by individuals with SEND, as research has already shown (Aishworiya & Kang, 2020), leading to more stressful and anxious feelings which in the long-term could lead to higher anxiety.

Understanding care givers mental health is also important; especially since it predicts anxiety of children and previous studies have shown that caregivers of children with SEND have additional stress and anxiety (Asbury et al., 2020; Ashworth et al., 2019). The current study provides evidence that further investigation is needed about this association as it can help understand the direction of this relationship between parent anxiety and child anxiety, but also subsequently how such high-quality of stress and anxiety impacts the parent–child as has been argued (Russell et al., 2020).

Finally, our data strongly suggest that both groups would benefit from schools and activity centres such the operation of these institutes during the current ongoing pandemic could be described as vital for now, but also for future lockdowns.

## Supplementary Information

Below is the link to the electronic supplementary material.

Supplementary file1 (DOCX 18 kb)
