# Supplementary material for: The Impact of COVID-19 on Anxiety and Worries for Families of Individuals with Special Education Needs and Disabilities in the UK
Source: J Autism Dev Disord. 2021 Jul 1;52(6):2656–69. doi: 10.1007/s10803-021-05168-5 (PMC8246131; doi:10.1007/s10803-021-05168-5)
Supplement: Supplementary file 1 — Supplementary file1 (DOCX 18 kb) [file 10803_2021_5168_MOESM1_ESM.docx]

**Table S1** Post Hoc Comparisons - Effect of Time on Anxiety for Children with SEND and TD Children

| **Post Hoc Comparisons - Time** | | | | | | | | | | |
| --- | --- | --- | --- | --- | --- | --- | --- | --- | --- | --- |
|  | | | **95% CI for Mean Difference** | |  | | | | | |
|  |  | **Mean Difference** | **Lower** | **Upper** | **SE** | **t** | | **Cohen's d** | ***p* _bonf_** | |
| Before | Start | -0.50 | -0.61 | -0.40 | 0.04 | -11.64 | -0.49 |  | < .001 | *** |
|  | Now | -0.81 | -0.92 | -0.71 | 0.04 | -18.83 | -0.79 |  | < .001 | *** |
| Start | Now | -0.31 | -0.41 | -0.21 | 0.04 | -7.19 | -0.30 |  | < .001 | *** |

Cohen’s d does not correct for multiple comparisons

P-value and confidence intervals adjusted for comparing a family of 3 estimates (confidence intervals corrected using the Bonferroni method)

Results are averaged over the levels of: Group

****p* < .001

**Table S2** Post Hoc Comparisons for Group: Anxiety for Children with SEND and TD Children

| **Post Hoc Comparisons - Group** | | | | | | | | | |
| --- | --- | --- | --- | --- | --- | --- | --- | --- | --- |
|  | | | **95% CI for Mean Difference** | |  | | | | |
|  |  | **Mean Difference** | **Lower** | **Upper** | **SE** | **t** | **Cohen's d** | ***p* _bonf_** | |
| SEND | TD | 0.38 | 0.17 | 0.59 | 0.10 | 3.62 | 0.15 | < .001 | *** |

Results are averaged over the levels of: Time

Cohen’s d does not correct for multiple comparisons

****p* <.001
